# Dust evolution, a global view: II. Top-down branching, nanoparticle fragmentation and the mystery of the diffuse interstellar band carriers

**DOI:** 10.1098/rsos.160223

**Published:** 2016-12-14

**Authors:** A. P. Jones

**Affiliations:** Institut d’Astrophysique Spatiale, CNRS, Univ. Paris-Sud, Université Paris-Saclay, Bât. 121, 91405 Orsay cedex, France

**Keywords:** interstellar dust, dust extinction, interstellar molecules

## Abstract

The origin of the diffuse interstellar bands (DIBs), one of the longest-standing mysteries of the interstellar medium (ISM), is explored within the framework of *The Heterogeneous dust Evolution Model for Interstellar Solids* (THEMIS). The likely nature of the DIB carriers and their evolution is here explored within the framework of the structures and sub-structures inherent to doped hydrogenated amorphous carbon grains in the ISM. Based on the natural aromatic-rich moieties (asphaltenes) recovered from coal and oil, the likely structure of their interstellar analogues is investigated within the context of the diffuse band problem. It is here proposed that the top-down evolution of interstellar carbonaceous grains, and, in particular, a-C(:H) nanoparticles, is at the heart of the formation and evolution of the DIB carriers and their associations with small molecules and radicals, such as C_2_, C_3_, CH and CN. It is most probable that the DIBs are carried by dehydrogenated, ionized, hetero-cyclic, olefinic and aromatic-rich moieties that form an integral part of the contiguous structure of hetero-atom-doped hydrogenated amorphous carbon nanoparticles and their daughter fragmentation products. Within this framework, it is proposed that polyene structures in all their variants could be viable DIB carrier candidates.

## 1. Introduction

The search for the origin of the diffuse interstellar bands (DIBs) has been a long journey (e.g. [1–3]); a journey that is far from over despite the recent assignment of four DIBs to the fullerene cation, C${\;}_{60}^{+}$ [4,5].

Here, we explore the fundamental nature and the very roots of the likely DIB carriers, and their formation, by profiting from the most recent atomic resolution analyses of aromatic moieties [6–9] and naturally occurring carbonaceous materials [10]. This work is also based on experimental and theoretical work on the dehydrogenation of polycyclic aromatic hydrocarbons (PAHs) to form cluster cations C_*n*_H${\;}_{x}^{+}$ with *x*≥0, e.g. [11–15].

The approach adopted here is generic^1^ and will, therefore, certainly not furnish the specific identification of a single DIB carrier. However, if this exploration can bring something to bear on the likely nature and evolution of solid carbonaceous matter and its relationship to the DIBs, especially at nanoparticle and smaller size-scales, then it could lead to the elucidation of whole families of related but structurally distinct species or grain sub-structures that may be central to an explanation for the DIBs, their origin, evolution and associations with particular molecules, ions and radicals.

This paper is structured as follows: §2 considers the challenge presented by the overwhelming variety of possible chemical structures; §3 proposes a new nomenclature scheme for simply characterizing hetero-aromatic moieties; §4 summarizes some of the known effects of dehydrogenation and ionization on aromatic species; §5 suggests a new form of hydrocarbon ring molecule; §6 investigates the likely nature of the DIB carriers in the interstellar medium (ISM); §7 proposes a new top-down branching route for nanoparticle evolution and the formation of the DIB carriers in the ISM; §8 suggests possible follow-on investigations and §9 concludes.

## 2. An embarrassment of riches?

For now, if we consider only hydrocarbon molecules, ions or radicals, with perhaps single O or N hetero-atoms (i.e. C_*n*_H_*x*_O or C_*n*_H_*x*_N species), we can simply characterize their contiguous structures by the number of multiply bonded (greater than or equal to 2), heavy atoms (C, O or N), which make up their chain and/or ring structures. For simple species containing up to six heavy atoms, i.e. C_6_H_*x*_, C_5_H_*x*_O or C_5_H_*x*_N with *x*≤(2*n*+2), table 1 shows an estimate of the number of structurally distinct species. The numbers in this table show the wide variety of possible chemical structures if we include only single O or N hetero-atoms, simple (de)hydrogenation/saturation, neutral and singly ionized species. Given that fully hydrogenated species are less likely to be ionized in denser regions, and ionized species are more likely to be dehydrogenated in ionized regions, we have conservatively divided the total number of possible species by two in order to try to estimate how many different configurations are likely to be of relevance to ISM chemistry. As table 1 shows, species with only six heavy atoms are likely to exhibit well over 1000 different structural forms. The number of possible conformations probably goes with, at least, the fourth power of the number of atoms. Hence, species with about 30 heavy atoms are likely to have more than a million possible structures. In any case, such small structures are probably not of significance because they will rapidly be destroyed in the diffuse ISM by ultraviolet and extreme ultraviolet (UV and EUV) photons [11,12,19] (hereafter referred to as UV) and only species with more than 50 carbon atoms are likely to survive [11,12]. Clearly, for a bottom-up chemistry investigation, as the number of atoms increases, a detailed study of all of the possible structures becomes intractable.

**Table 1.** Hydrocarbon chemical complexity in the ISM. In column 1, the prefix c- indicates that a stable cyclic form exists *in addition* to the chain and branched-chain isomers. The approximation of the number of most probable different forms is given by (*i*×*n*×*b*×*h*×[2*n*+3])/2. The dehydrogenated states (column 5) does not count the number of different configurations possible for each *n* and is therefore only the lower limit.

structure (*p*=0 or 1) {charge states, *i*=2}	no. of atoms (*n*)	no. of isomers (*b*)	no. of hetero-atomic isomers (*h*) [cyclic form only]	minimum no. of (de)hydrogenated states (2*n*+3)	≈ no. of possible forms
(CX_*p*_) {0/+}	1	1	1 [0]	5	5
(CX_*p*_)_2_ {0/+}	2	1	1 [0]	7	14
c-(CX_*p*_)_3_ {0/+}	3	2	2 [2]	9	108
c-(CX_*p*_)_4_ {0/+}	4	2	2 [0]	11	176
c-(CX_*p*_)_5_ {0/+}	5	4	3 [1]	13	780
c-(CX_*p*_)_6_ {0/+}	6	6	3 [1]	15	1620

## 3. Polycyclic (hetero-)aromatic structure characterization and nomenclature

Here, we explore and characterize the basic carbonaceous material (sub-)structures occurring in natural materials (petroleum and coal asphaltenes) with the view that these same types of structures ought also to be present in carbonaceous grains in the ISM.

### 3.1 Structure nomenclature

To characterize carbonaceous frameworks, a new nomenclature scheme is proposed here to enable a general description of asphaltene-type structures and in order to provide a descriptor for the likely sub-structures in interstellar carbonaceous grains. Specifically, for the intrinsic five-, six- and seven-membered rings, we adopt the following labels:

P = pentagonal, fivefold rings
S = hexagonal, sixfold rings
G = heptagonal, sevenfold rings.

To enable the maximum flexibility in the use of aromatic structure descriptor, the following rules and indicators are proposed:

— The naming sequence begins with the largest S aromatic domain, unless this is a central moiety.
— A subscript *n*, e.g. S_*n*_, indicates the total number of rings (more than one) of the given type within any distinct domain but does not consider the particular arrangement of the rings.
— Distinct ring domain names are concatenated, e.g. S_*n*_P_*m*_, in the same arrangement as they appear in the structure.
— A superscript ^′^ indicates a methylene (–CH_2_–) bridge in the given ring, e.g. P′ or S′.
— A superscript^X^ indicates a hetero-cycle containing one (two) hetero-atoms, e.g. P^X^ (P^XX^), where X=N, O, S, Si, P, B, …
— Pendant S or P rings attached to the larger structure by a single bond are indicated by –S or –P, respectively.

Table 2 shows some simple examples of how this nomenclature scheme can be applied to some simple aromatic and polycyclic aromatic species.

**Table 2.** Polycyclic (hetero-)aromatic nomenclature.

name	formula	descriptor
benzene	C_6_H_6_	S
naphthalene	C_10_H_8_	S_2_
azulene	C_10_H_8_	GP
biphenyl	C_12_H_10_	S–S
fluorene	C_13_H_10_	SP′S
anthracene	C_14_H_10_	S_3_
phenanthrene	C_14_H_10_	S_3_
pyrene	C_16_H_10_	S_4_
2,3-benzofluorene	C_17_H_12_	S_2_P′S
benz[a]anthracene	C_18_H_12_	S_4_
chrysene	C_18_H_12_	S_4_
triphenylene	C_18_H_12_	S_4_
corannulene	C_20_H_10_	S_5_P
perylene	C_20_H_12_	S_5_
benzo[ghi]perylene	C_22_H_12_	S_6_
coronene	C_24_H_12_	S_7_
fullerene^*a*^	C_60_	S_20_12×P

^*a*^In fullerene, the 12 pentagons are isolated, hence, the descriptor should be S_20_PPPPPPPPPPPP, but this is rather cumbersome and so a simplified 12×P descriptor is used above.

### 3.2 Asphaltenes: a framework guide

Here, we explore the structure of asphaltenes, which are natural, aromatic-rich species derived from petroleum (PA) and coal (CA). More than 100 of these PA and CA asphaltene species were recently analysed by atomic force microscopy (AFM) and molecular orbital imaging using scanning tunnelling microscopy at atomic resolution [10]. In the following, we use the results of this study [10] as a basis for the discussion presented in this section.

Both PA and CA asphaltenes show a wide range of complex aromatic structures, often with attached methyl and large alkyl peripheral substitutions. While sixfold aromatic rings are predominant in the analysed structures, they also contain a significant fraction of fivefold rings, rare sevenfold rings and apparently no three- or fourfold rings. No two identically structured asphaltenes were observed in the experiments and the structures are often non-planar. Among the analysed species, organic radicals (CA12), charged species (CA3) and even an 18 ring ‘nano-graphene’ (CA6) were observed [10] (table 3).

**Table 3.** Some typical asphaltene structures ordered by an increasing number of carbon atoms in ring structures, N_*C*_, the number of rings, N_*R*_, and the ring variety, S → SP → GSP. The lines separating species with more than 30 C atoms from those with fewer indicate the approximate limit for the size of the aromatic-rich species in the diffuse ISM (e.g. [19]).

asphaltene specimen	N_*C*_	N_*R*_	C atoms per ring N_*C*_/N_*R*_	methyl side groups (–CH_3_)	alkyl side groups (–R)	descriptor
S systems (sixfold rings)						
CA36	22	5	4.4	1	2	S_4_–S
CA74	22	6	3.7	0	1	S_6_
CA49	24	6	4.0	0	2	S_4_S^*X*^S
CA12	25	6	4.2	1	0	S_6_
CA47	25	7	3.6	0	1	S${\;}_{7}^{X}$
CA44	25	7	3.6	1	0	S_7_
CA22	25	7	3.6	0	0	S${\;}_{7}^{X}$
CA65	26	7	3.7	0	0	S_7_
CA1	28	8	3.5	1	0	S_8_
CA9	28	8	3.5	1	0	S_8_
CA61	28	8	3.5	0	0	S_8_
CA14	30	9	3.3	1	0	S_9_
CA34	32	7	4.6	0	1	S_4_–S_3_
CA7	32	9	3.6	1	0	S_9_
CA79	34	10	3.4	1	3	S_10_
CA5	35	11	3.2	0	2	S_11_
CA72	40	11	3.6	0	3	S_11_
CA6	58	18	3.2	0	0	S_18_
SP systems (six- and fivefold rings)						
CA21	18	5	3.6	0	0	S_4_P^*X*^
CA23	20	5	4.0	2	0	SP^*X*^P^*X*^S–S
CA41	20	5	4.0	0	1	S_3_P^*X*^S
CA95	20	6	3.3	2	0	S${\;}_{5}^{X}$P
CA54	30	8	3.8	1	2	S_6_P^*X*^S
PA3	30	9	3.3	1	2	S_3_P^*X*^P^*X*^S_2_P^*X*^S
CA48	31	9	3.4	1	0	S_6_S^*X*^S^*X*^S
CA2	34	9	3.8	1	0	S_4_PS_4_
CA13	34	9	3.8	0	0	S_7_PS
CA39	40	8	5.0	1	0	S_6_P^*X*^S
CA91	42	12	3.5	0	4	S_11_P^*XX*^
CA16	44	11	4.0	0	0	S_3_P^*X*^S_3_–S_4_
CA60	46	14	3.3	0	4	S_9_PS_2_P^*X*^S
CA15	54	15	3.6	0	0	S_3_PS_8_P^*X*^S_2_
GSP systems (seven-, six- and fivefold rings)						
CA3	40	12	3.3	3	0	S_5_P′GPS_2_P′S

Notes: The number of C atoms in rings ignores pendant alkyl side groups (–R) such as –CH_3_.

P′ indicates fivefold (P) ring sites with single methylene, –CH_2_– substitutions.

S^*X*^ and P^*X*^ (P^*XX*^) indicate peripheral sixfold (S) and fivefold (P) ring sites with single (double) hetero-atom substitutions (i.e. hetero-cycles).

The asphaltene PA3 is from virgin crude oil and did not undergo further treatment, all the other analysed asphaltenes (CAs) are derived from coal.

Typically, asphaltenes exhibit a central aromatic core with peripheral alkane chains, in some cases the aromatic cores comprise several distinct PAHs connected by single bonds. Nevertheless, the most common type of structure is a single aromatic core with often long alkane side groups. Among the analysed species [10], there is no sign of any cage-like structures, probably because they contain very few C atoms, i.e. *N*_C_<60, and are, therefore, significantly smaller than fullerenes. It has been noted that molecular re-arrangements often occurred during the AFM measurements making an attribution of the structure of the non-planar side groups particularly difficult, nevertheless methyl (–CH_3_) appears to be the most abundant side group [10]. Furthermore, in these experiments, it was not possible to unambiguously determine the nature of the hetero-atoms/moieties in the P rings, which could be CH, CH_2_, CO, N, NH or S.

Using as a reference the superbly detailed analytical work on the structure of over 100 asphaltene species [10], and as summarized in table 3 using the above nomenclature (§3.1), we can derive some general observations about the types of aromatic-rich sub-structures likely to be found in asphaltenes:

— Relatively compact (peri-condensed) structures dominate.
— Very extended (cata-condensed) structures are not common.
— Some show PAH islands, S_*n*=1−3_, linked by single bonds.
— PAs show more ring substitutions and P rings than CAs.
— PAs exhibit longer side groups than CAs.
— PA side groups can be up to approximately 2 nm long (≲15 C–C bonds).
— Central core S_*n*_ domains can be up to approximately 20 rings, i.e. ≲S_20_.
— For most S_*n*_ domains, *n* is typically ≃4–10 rings.
— S_*n*_ domains show no methylene (–CH_2_–) bridges.
— S_*n*_ domains are about as common as mixed S_*n*_P_*q*_(G) species.
— P rings are generally single and bridging, e.g. S_*n*_PS_*m*_.
— P rings can have methylene (–CH_2_–) bridges, i.e. P′.
— Most P rings are peripheral hetero-cycles, i.e. P^X^.
— P hetero-cycles are more abundant than S hetero-cycles.
— P^X^ systems can be paired, i.e. P^X^P^X^.
— Double hetero-atom cycles are seen, i.e. P^XX^.

In the experiments, it was noted that the conformation of the analysed asphaltenes was affected by their adsorption on the surface, which tends to force them into a more planar configuration than the results would indicate [10]. Also, sixfold ring aromatic-dominated structures, S_*n*_, are probably more easily analysed in the data and therefore may be somewhat over-represented in this large but still somewhat limited asphaltene sampling. Among the illustrated structures, there does not appear to be a preference for any particular size of the S_*n*_ domains, i.e. all values of *n* from 1 to 9 appear equally probable.

In table 3, the characteristic structures of the analysed CA asphaltenes [10] are described using the nomenclature scheme proposed above (§3.1). From this table, the preponderance of the S-rich aromatic moieties is clear, as is the bridging role of the P rings and their hetero-cyclic nature.

Using the ideas gleaned from the (sub-)structures observed in the naturally occurring aromatic-rich asphaltenes (table 3), we propose that similar sub-structures are also likely to be present in [X-doped] interstellar hydrogenated amorphous carbon, a-C(:H[:X]), grains and especially in aromatic-rich a-C nanoparticles. We now explore the likely consequences of the presence of such sub-structures in nanoparticles in the ISM.

## 4. Dehydrogenated and ionized aromatic structures

It has long been proposed in astrophysical studies that interstellar aromatic species will be dehydrogenated and ionized under the harsh UV irradiation conditions of the diffuse ISM. However, it has been found that ionized and partially dehydrogenated aromatic species are less resistant to photo-destruction than the parent neutral molecules. For example, the ovalene cation, C_32_H${\;}_{14}^{+}$, has a photo-dissociation rate that is four times that of the neutral molecule and for the completely dehydrogenated form, C${\;}_{32}^{+}$, it is three orders of magnitude larger [12]. From this work, it appears that only ‘PAHs’ with *n*_C_<50 would be significantly dehydrogenated. However, these same ‘PAHs’ will be destroyed by C_2_H_2_ loss on a time-scale of years in regions of the ISM with high UV radiation field strengths (such as reflection nebulae, planetary nebulae and HII regions) where the IR emission bands are observed [11].

However, the consequences of the loss of hydrogen atoms from aromatic carbonaceous species and the structure of the resulting radical ion have rarely been considered. It generally seems to be assumed that aromatic species, such as PAHs, retain their aromatic network structures and just lose hydrogen atoms from the periphery, without any structural re-arrangement. Furthermore, it is assumed that hydrogen atom addition to the radical ion re-generates the original aromatic structure. However, in the case of severely dehydrogenated PAHs, this view is probably rather simplistic and seems to be contradicted by experimental evidence (e.g. [15]).

The stepwise dehydrogenation of a suite of PAHs^2^ to form C_*n*_H${\;}_{x}^{+}$ cations has been extensively studied using sustained off-resonance irradiation [15]. In these experiments, the irradiation resulted in the sequential loss of single hydrogen atoms from the parent ions and the eventual formation of completely dehydrogenated carbon cluster ions, C${\;}_{n}^{+}$, in the case of coronene, perylene and benz[a]anthracene. En route to the formation of C${\;}_{24}^{+}$, from C_24_H${\;}_{6}^{+}$, no intermediate C_*n*_H${\;}_{x}^{+}$ cations with *n*=1, 2 or 4 were formed, and about two-thirds of the C_24_H${\;}_{3}^{+}$ cations decompose through carbon atom and hydrocarbon fragment loss. For smaller PAHs, no carbon cluster ions were observed, most probably because of fragmentation. In the course of the experiments, it was noted that C_*n*_H${\;}_{5}^{+}$ ions could be formed after the loss of five hydrogen atoms from pyrene and fluorene or seven hydrogen atoms from coronene, perylene, benz[a]anthracene, chrysene and triphenylene. The loss of further hydrogen atoms to form C_*n*_H${\;}_{4}^{+}$ or C_*n*_H${\;}_{3}^{+}$ ions was only seen for benz[a]anthracene, chrysene and triphenylene. Different dissociation pathways to C_*n*_H${\;}_{x}^{+}$ ions with odd and even numbers of hydrogen atoms were noted, with even electron species C_*n*_H${\;}_{2m+1}^{+}$ dissociating more easily, via single hydrogen atom loss, to form more stable C_*n*_H${\;}_{2m}^{+}$ ions. The difficulty in forming C_*n*_H${\;}_{x}^{+}$ (0≤*x*≤4) by stepwise hydrogen atom elimination is assumed to be due to significant differences in the structure of cation radicals with less than five hydrogen atoms [15].

### 4.1 Dehydrogenation pathways for aromatic moieties

The dehydrogenation of molecular PAH ions most probably proceeds by the elimination of two immediately adjacent (ortho) hydrogen atoms leading to a stable aryne bond. The aryne bond was long assumed to be acetylenic in nature (e.g. [20]). However, more recent work, pointed out in an article by Philip Ball entitled ‘First snapshot of elusive intermediate’ [21] highlights the results of the work reported in [6–8]. Using atomic resolution imaging, these researchers have shown that the aryne bond is actually a nonlinear C=C=C=C ‘cumulene-like’ structure.^3^

**Figure 1.**
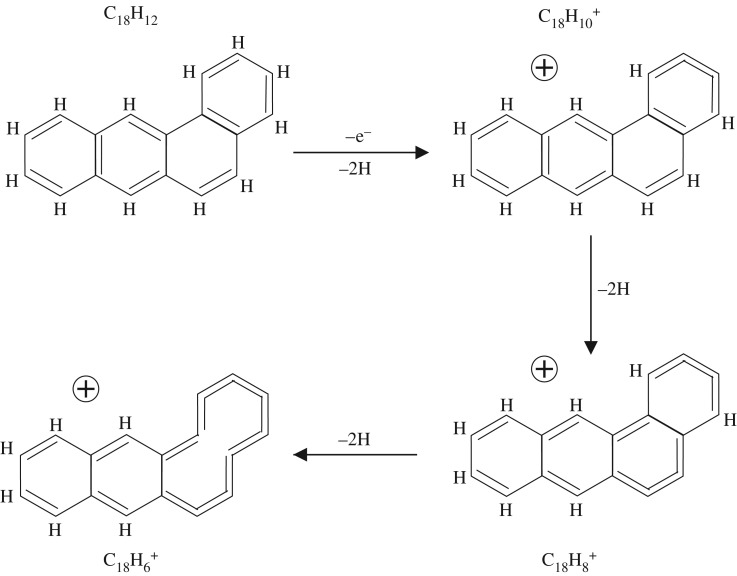
A proposed pathway for the dehydrogenation of C_18_H_12_ (S_4_) benz[a]anthracene to C_18_H${\;}_{6}^{+}$. A further single hydrogen atom dehydrogenation would then form a C_18_H${\;}_{5}^{+}$ ion.

**Figure 2.**
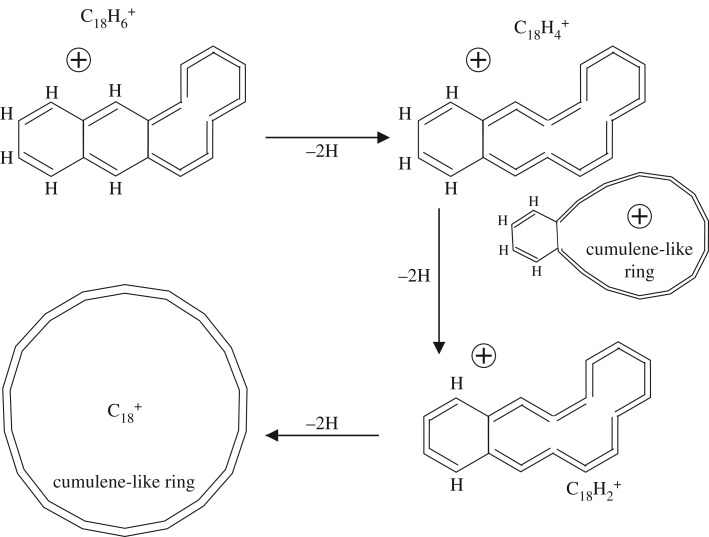
A proposed pathway for the dehydrogenation of the C_18_H${\;}_{6}^{+}$ cation to C${\;}_{24}^{+}$. The double ring structure in the upper and middle right shows the most probably bicyclic form of the C_18_H${\;}_{4}^{+}$ cation, i.e. an ortho-substituted sixfold ring with a 12-carbon atom cumulene-type ring [13–15].

For the C_18_H_12_ PAHs benz[a]anthracene, chrysene and triphenylene, dehydrogenation proceeds by a similar route and leads to the bicyclic product, C_18_H${\;}_{4}^{+}$, which is apparently more stable than linear and single-cycle species [14]. The most stable bicyclic product is thought to contain a benzene ring ortho-substituted by a carbon loop [13,14]. Figure 1 shows a likely dehydrogenation sequence for the C_18_H_12_ PAH benz[a]anthracene to the C_18_H${\;}_{6}^{+}$ ion, which proceeds via the progressive dehydrogenation of the three-ring phenanthrene sub-structure to form the cumulene-like structures noted above. Chrysene and triphenylene also contain the phenanthrene sub-structures and so the progressive dehydrogenation of these molecules to C_18_H${\;}_{6}^{+}$ ions will most likely follow the same sequence to the same product ion. In each of these cases, the C_18_H${\;}_{6}^{+}$ ion has been dehydrogenated to a three-ring structure through cumulene-like chain formation, i.e. the formation of double-bonded C=C=C=C chains and eventually a 10 carbon atom cumulene-like chain (lower left structure in figure 1). The problem with this scenario is that it does not easily explain why the C_18_H${\;}_{6}^{+}$ ion structure is somewhat special because sixfold ring break-up has already started. The further dehydrogenation of this form of the cation could, in any event, proceed along the pathways shown in figure 2 to yield the product bicyclic C_18_H${\;}_{4}^{+}$ ion [13,14] and to a possible structure for the C${\;}_{18}^{+}$ carbon cluster. Indeed, this type of radicalized double aromatic ring-opening transformation, well known as Bergman cyclization, was recently revealed in its full structural glory through a beautiful scanning tunnelling microscopy experiment [9].

**Figure 3.**
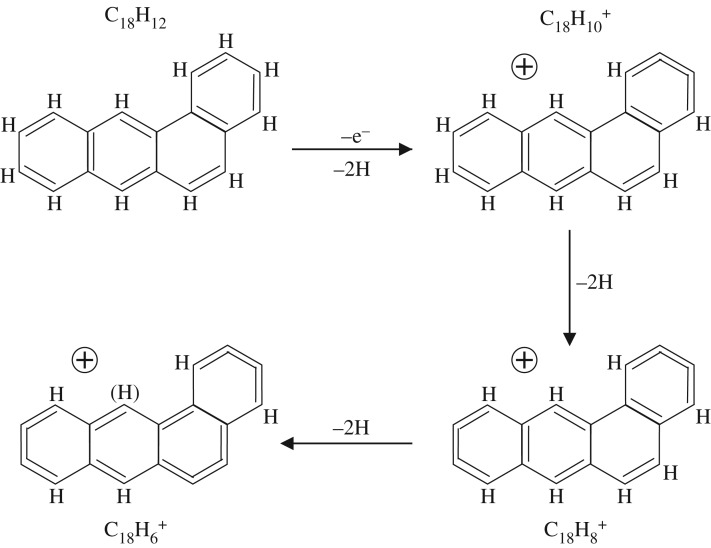
An alternative pathway for the dehydrogenation of C_18_H_12_ (S_4_) benz[a]anthracene to C_18_H${\;}_{6}^{+}$. The bracketed hydrogen atom, (H), in the lower left ion indicates a site than could be dehydrogenated to form a C_18_H${\;}_{5}^{+}$ ion.

**Figure 4.**
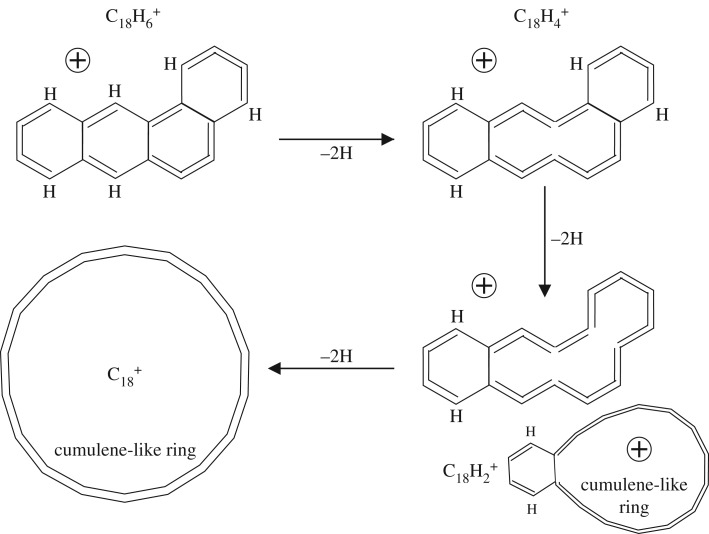
The follow-on to the alternative dehydrogenation pathway for the C_18_H${\;}_{6}^{+}$ cation to C${\;}_{18}^{+}$. In this case, the formation of a different bicyclic cation, C_18_H${\;}_{2}^{+}$, occurs somewhat later in the dehydrogenation sequence.

It appears that an alternative pathway involving the preferred and sequential formation of C=C=C=C cumulene-like structures first may also provide a viable route to the C_18_H${\;}_{6}^{+}$ cation (figure 3) without the need to break sixfold rings. In this case, the dehydrogenation has seemingly gone about as far as it can go along this path without a major disruption of the original PAH structure. In this case, a critical-state ion structure has been formed, for which the further dehydrogenation of this C_18_H${\;}_{6}^{+}$ cation (lower left structure in figure 3) can proceed only via the formation of cumulene-like chains through the disruption of sixfold ring structures. Figure 4 shows a likely dehydrogenation sequence for a cumulene-dominated form of the benz[a]anthracene-derived ion C_18_H${\;}_{6}^{+}$ to an alternative product bicyclic structure, C_18_H${\;}_{2}^{+}$, and to the same form of the C${\;}_{18}^{+}$ carbon cluster as for the previously described pathway shown in figures 1 and 2. Using simple bond energy considerations, i.e. summing the energy due to the breaking of C–H bonds and the conversion of C–C bonds into C=C bonds, it can be shown that the alternative pathway to the formation of the C_24_H${\;}_{6}^{+}$ cation (figure 3) appears to be more favourable by almost 4 eV.

Regardless of the pathway that PAH dehydrogenation actually follows, figures 1–4 show that significant changes in structures are to be expected. In particular, the pathway shown in figures 3 and 4 shows a major structural difference between the cations with five or six hydrogen atoms (figure 3) and those with less than 5 hydrogen atoms (figure 4), which could perhaps better explain the difference in the dehydrogenation behaviour of these cations. However, in this case, the products formed prior to complete dehydrogenation are a tricyclic C_18_H${\;}_{4}^{+}$ ion or a bicyclic C_18_H${\;}_{2}^{+}$ ion rather than a bicyclic C_18_H${\;}_{4}^{+}$ cation [13–15].

Nevertheless, and whatever the exact dehydrogenation route, it seems that in all cases the dehydrogenation of PAH-like structures proceeds by single hydrogen atom elimination down to C_*n*_H${\;}_{5}^{+}$ but thereafter involves significant carbon atom loss from the structure, i.e. by a fragmentation of the parental PAH framework. In some cases (C_*n*_H${\;}_{x}^{+}$; *n*≥20, *x*≤10), the cation species do perhaps retain a memory of the parent PAH, suggesting that some PAH structures may be only slightly modified by low-energy hydrogen elimination [15]. Thus, it appears that the exact routes for PAH dehydrogenation and cation formation are particularly dependent upon the details of the aromatic structure and perhaps the symmetry of the parent PAH, with highly symmetric PAHs following a different path from their asymmetric and cata-condensed sisters.

It has been found that adding hydrogen to C${\;}_{n}^{+}$ carbon cluster cations, with *n*=9 to 22, leads to a change in the structure from monocyclic C${\;}_{n}^{+}$ to linear C_*n*_H${\;}_{x}^{+}$ forms (*x*≤3) [13,14]. In addition, a new but minor bicyclic isomer appears from *x*=4 and *n*≥15, which increases in abundance with *n* at the expense of the monocyclic and linear forms. Furthermore, the bicyclic forms of the C_16_H${\;}_{4}^{+}$ ion appear to be much more stable than either the monocyclic or linear forms [14].

**Figure 5.**
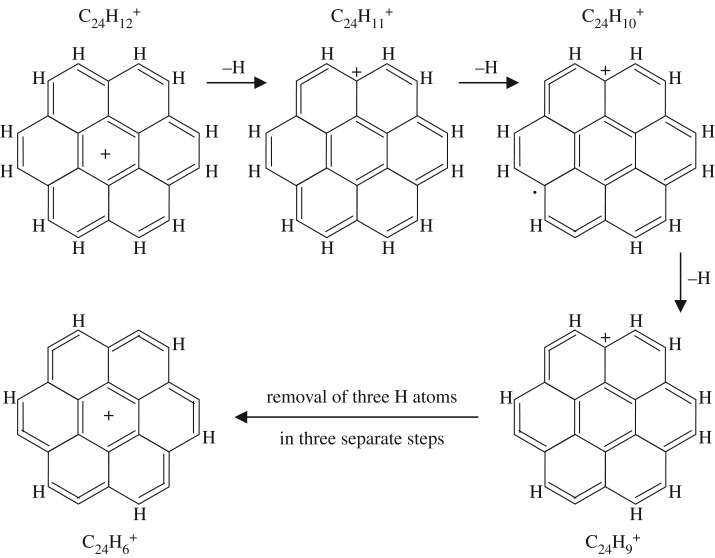
One possible pathway for the dehydrogenation of the C_24_H${\;}_{12}^{+}$ (S_7_) coronene cation to C_24_H${\;}_{6}^{+}$.

**Figure 6.**
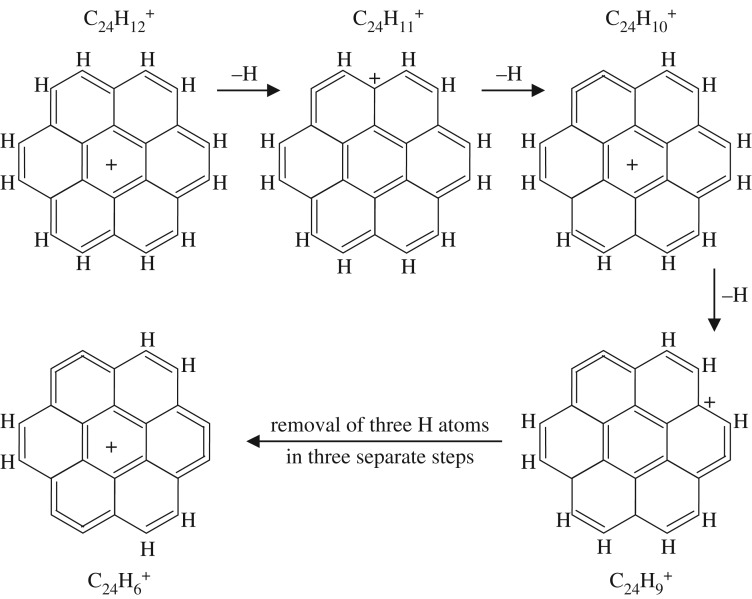
A second possible pathway for the dehydrogenation of the C_24_H${\;}_{12}^{+}$ (S_7_) coronene cation leading to C_24_H${\;}_{6}^{+}$ structure with a different but equally symmetric structure.

**Figure 7.**
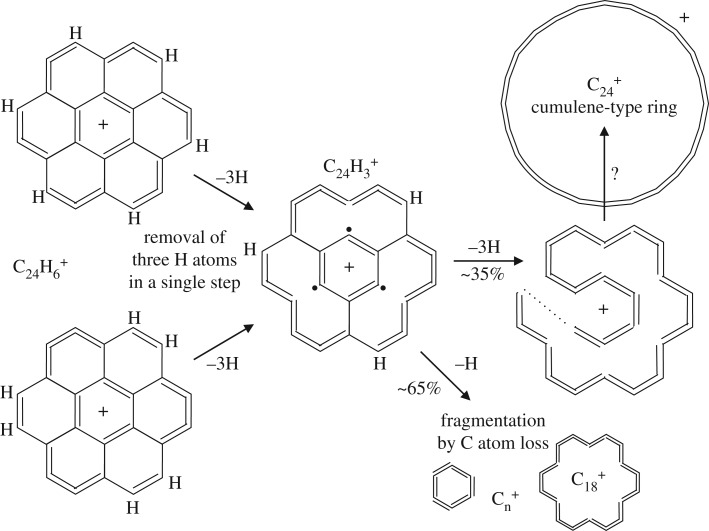
A possible pathway for the dehydrogenation of the C_24_H${\;}_{6}^{+}$ ion leading to C${\;}_{24}^{+}$, which is assumed to have a cumulene-type ring structure.

The dehydrogenation of the larger coronene molecule must also follow a similar sequence but with slight differences indicated by the inability to form certain configurations along the way [15]. Figures 5 and 6 show possible pathways for the single H atom stepwise dehydrogenation of the coronene cation C_24_H${\;}_{12}^{+}$ to C_24_H${\;}_{6}^{+}$. The two pathways lead to equally symmetric cations but to quite different structures. In both of the suggested pathways, and up to this point, the seven-ring structure of the parent coronene molecule is preserved. The further dehydrogenation of the C_24_H${\;}_{6}^{+}$ cation can proceed only by the breaking of one of the sixfold rings. As mentioned above, the formation of the carbon cluster C${\;}_{24}^{+}$, by the dehydrogenation of the C_24_H${\;}_{6}^{+}$ ion, forms only an intermediate C_24_H${\;}_{3}^{+}$ cation and a large fraction ($∼\frac{2}{3}$) of these ions actually fragment en route to C${\;}_{24}^{+}$ [15]. Thus, the intermediate species with *x*=1, 2 or 4 are either highly unstable or are bypassed by the dehydrogenation-imposed re-structuring, i.e. the simultaneous breaking of three sixfold ring bonds to form C_24_H${\;}_{3}^{+}$ and then a further four sixfold ring bonds. Figure 7 shows a likely scenario for the C_24_H${\;}_{x}^{+}$ (*x*<6) cation re-structuring leading to C_24_H${\;}_{3}^{+}$ and ultimately C${\;}_{24}^{+}$ during dehydrogenation.

### 4.2 Some consequences of dehydrogenation

The re-structuring of aromatic moieties during dehydrogenation, via cumulene-like chain and ring formation could have some interesting consequences for the UV photo-processing of the sub-structures in a-C(:H) nanoparticles in photo-dissociation regions (PDRs) and HII regions. Clearly the aromatic sub-structures intrinsic to the grains can be dehydrogenated, as described above, but following a recently proposed H_2_ formation mechanism it is most likely that the aliphatic/olefinic bridges between the aromatic moieties are UV processed before the aromatic domains are destroyed [22]. This work also suggests that the UV processing of the bridges can take them as far as olefinic species, but it now seems that their complete dehydrogenation could result in cumulene-like structures that will most likely be formed just before or in the process of nanoparticle fragmentation.

Hydrogen atom addition to a cumulene-type ring is known to lead to linear species, i.e. ring-breaking [13,14]. Thus, it is likely that the severe dehydrogenation of aromatic species in the ISM leads to structures that are very different from the parent structure. The reaction of gas-phase hydrogen atoms with these dehydrogenated cyclic or bridging species probably does not necessarily lead to the re-formation of the original structure, through re-hydrogenation, but rather to linear molecule formation and/or the fragmentation of the chains.

In the following, some of the consequences of UV processing in intense radiation field regions leading to cumulene-type structure formation are considered. The following schematic indicates how cumulene-like bridging species, some with nitrogen hetero-atoms, could form and how they might react with atomic hydrogen (the ‘dangling’ bonds illustrated as –, = and ≡ are assumed to be connected to the larger contiguous grain structure):

4.1 $$\begin{array}{rl} & -{\mathrm{CH}}_{2}-{\mathrm{CH}}_{2}-{\mathrm{CH}}_{2}-{\mathrm{CH}}_{2}-\;\;\;\;-\;4H\;\;\;\to \\ & -\mathrm{CH}\text{=}\mathrm{CH}-\mathrm{CH}\text{=}\mathrm{CH}-\;\;\;\;\;\;\;\;-\;4H\;\;\;\to \\ & \text{=}C\text{=}C\text{=}C\text{=}C\text{=}\;\;\;\;\;\;\;+\;\;\;H\;\;\;\to \;\;\;H-C\equiv C-C\equiv C-\\ & \text{=}C\text{=}C\text{=}N-\mathrm{CH}\text{=}\;\;\;+\;\;H\;\;\;\to \;\;\;\equiv C-C\equiv N+H_{2}C\text{=} \end{array}$$

The above sub-structures could be parts of cumulene-type rings or the dehydrogenated bridging links between the aromatic domains in a-C(:H) nanoparticles. Perhaps, these species and their reaction with atomic hydrogen could provide an alternative top-down route to (cyano)polyyne molecule formation in the ISM.

## 5. A new form of carbon molecule

**Figure 8.**
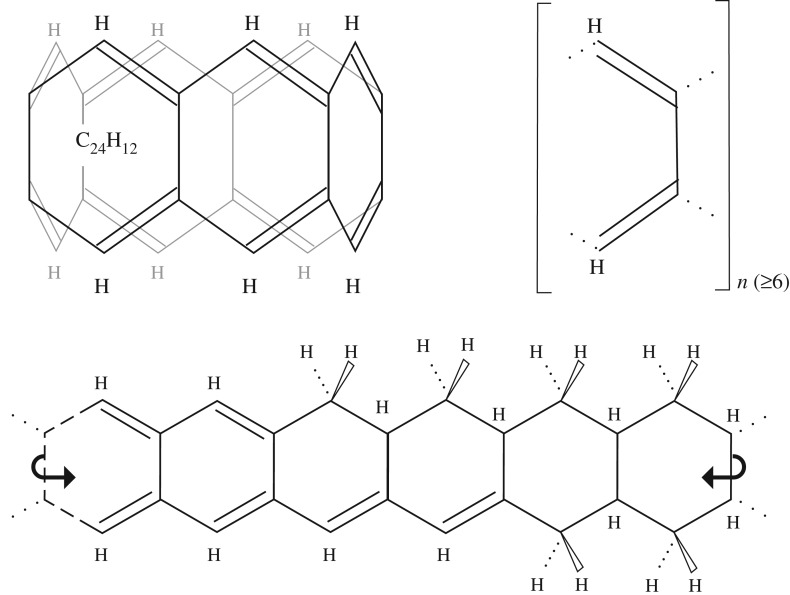
A proposed ‘ring of rings’ family of molecules: couronene, C_24_H_12_ (S_6_, upper left), the structure for a concatenated cycle of aromatic rings with generic formula (C_4_H_2_)_*n*_ (S_*n*_, upper right). If all the carbon atoms are *sp*^3^, then the aliphatic multi-ring structure is a couronane, C_24_H_36_, generically (C_4_H_6_)_*n*_, where *n*≥6. Mixed aliphatic/aromatic ring structures would then be courone-anes, with the generic formula (C_4_H_2−6_)_*n*_, where *n*≥6.

A coronene isomer and a new family of aromatic structures (C_4_H_2_)_*n*_, with *n*≥6 is proposed; the name couronenes is suggested for these molecules because of their crown-like structure. These structures are, in fact, carbon nano-tubes with a cylinder length of only one sixfold ring. However, they form a closed ring rather than the spiral structure often found in carbon nano-tube structures. Couronenes could be particularly interesting because the upper and lower crown edges are always stable annulene structures, which can probably be hydrogenated to form aliphatic couronanes (C_4_H_4_)_*n*_, with *n*≥6, or mixed couronenes/couronanes (C_4_H_3_)_*n*_, with *n*≥6 (courone-anes), with upper and lower crown edges having olefinic and aliphatic structures, respectively, or mixed olefinics and aliphatics on each edge. Figure 8 shows some examples of the likely structures of this proposed family of crown-like molecules. These species ought to have tuneable properties because, in principle, any number of rings can be joined in this way. Although there must be some sterically determined minimum ring number limit, which could mean that the minimum couronene/couronane molecules contain no less than 24 carbon atoms, i.e. *n*≥6. Seemingly, the addition of one or more nitrogen atoms into the structure, in a number of possible structural isomers, might lead to some interesting transitions in the visible.

Furthermore, if the upper and lower edges of the crown can each be closed with a cap composed of a sixfold ring and six accompanying pentagons, then the result will be a closed C_36_ fullerene structure.

How might such couronene species be formed in the ISM, if indeed they are present there? The most likely route would be in the same inefficient way that fullerenes are formed, i.e. via the UV photon interactions that lead to the vibrational excitation and the photo-processing dehydrogenation of a-C(:H) nanoparticles [23]. However, as for fullerenes, this route to their formation is likely to be equally inefficient because most of the possible parent species will bypass these stable configurations and therefore only a small fraction of the carbon in dust in the ISM could be incorporated into couronenes, if they even exist there.

However, independent of whether courone-ane species exist in the ISM, their intrinsic properties are perhaps sufficiently interesting enough to warrant the further exploration of this idea.

## 6. The nature of the diffuse interstellar band carriers

Given that we have yet to find an explanation for more than 99% of the DIBs, it would seem that some theoretical considerations, guided by experimental results, might yet be able to bring something to bear on the DIB problem by providing ideas about subsets of likely species that could then be explored in the laboratory. With this aim in mind, this section aims to provide a framework to elucidate the likely fundamental nature of the DIB carriers.

### 6.1 The observed properties of the diffuse interstellar band carriers

Here, a brief and cursory summary of the major observational constraints on the DIBs is given. The interested reader should refer to the most recent proceedings and the more recent literature on the subject for a more complete overview of the subject (e.g. [1,3]).

There are over 400 known and well-determined DIBs, and of these approximately 100–200 are common bands, i.e. they are observed along every line of sight where the DIBs are seen, and they show a wide range of widths and intensities. For the handful considered so far, strong DIBs are not polarized. There do appear to be families of bands that are similar from one line of sight to another, but these are very generic.

The observational aspects of the DIBs presented here were drawn from an obviously limited selection of the enormous literature on the subject (e.g. [1,24–35]). In general, several key correlation and trends between the DIBs and environmental factors are seen, including that

— strong DIBs trace the diffuse atomic, HI, gas,
— they show a positive correlation with *N*_HI_,
— the show all possible correlation behaviours with *N*_H_2__,
— they are associated with the diffuse rather than the dense ISM,
— they are broader than atomic or molecular lines,
— they are weaker in strong UV radiation fields,
— they show varied stability to UV radiation,
— broader DIBs are less sensitive to UV radiation,
— some NIR DIBs correlate and may be due to cations,
— inter-DIB correlations do not always go through zero, i.e. some DIBs are (compositionally) inter-dependant,
— they generally do not correlate well with each other,
— they correlate with dust, i.e. in extinction, *E*(*B*−*V*), and emission, which is itself a proxy for *N*_H_(total),
— they show a weak negative correlation with the 217 nm UV bump,
— they show a weak negative correlation with the FUV extinction,
— weak DIBs correlate with enhanced FUV extinction,
— they show a ‘skin’ effect, i.e. strength decreasing above some *E*(*B*−*V*) value,
— they correlate with small molecules/radicals (but less well than with dust),
— some appear to be somehow associated with, for example, C_2_, C_3_, CN, CH,
  but the same DIBs can be seen both with and without them,
— very few DIBS (approx. 12 DIBs, i.e. less than 5%) show PQR branches, perhaps indicating prolate top molecules with ≲5–7 atoms.

These characteristics seem to strongly indicate that the DIB carriers are most likely formed by a top-down fragmentation process because a formation by bottom-up chemistry would lead to the same chemistry along all lines of sight.^4^ However, the same DIBs are observed along lines of sight with and without the same molecules, radicals and ions. For example, in a study of 414 DIBs in the λ=390–810 nm region towards HD 183143, it was found that the DIBs are redder, broader and weaker and that there is no C_2_, whereas the line of sight towards HD 204827 shows the same DIBs but high *N*(*C*_2_)/*E*(*B*−*V*) [26]. However, there appear to be weak features along both lines of sight and most of the DIBs are present towards both stars, albeit with very different relative strengths.^5^

Interestingly, PAH cations are known to absorb in the visible and near-IR, but no neutral PAHs or PAH cations have yet been detected in diffuse ISM and such species cannot, therefore, be the carriers of the DIBs (e.g. [40–42]). This is consistent with work that shows that neutral and ionized PAHs cannot be the carriers of DIBs [43]. Furthermore, it has been shown that *l*-C_3_H_2_ is not a DIB carrier and it is therefore likely that this and other small linear molecules are not the origin of the DIBs [31].

### 6.2 Theoretical constraints on the diffuse interstellar band carriers

The DIBS are almost certainly due to the electronic transitions of the carrier species, which could be molecules, polyatomic radicals and/or ions. As has clearly been shown, the 578.0 nm DIB towards Hershel 26 is most probably due to a polar molecule with n≲7 [32]. Thus, small five to seven heavy atom polar species are the most probable culprits for some DIBs, at least for those that show PQR-type branch structures. Perhaps, if the carrier species of the 578.0 nm DIB are cyclic, then they could contain about double the number of atoms (i.e. 10–15 heavy atoms). The conundrum here is that such small species are likely to have a rather short lifetime in the diffuse ISM, where only (aromatic-rich) species with more than about 30–50 carbon atoms appear to be stable [12]. However, this may not be a hard limit because smaller species could also be DIB carriers if they exhibit an intrinsic stability against UV photo-dissociation due to efficient cooling processes, such as inverse internal conversion resulting in optical photon emission. This question is explored in more detail in the following §6.5.

It was recently proposed that carbonaceous nanoparticles could provide a viable explanation for the DIBs [44]. In this case, the hetero-atom doping of a-C(:H) (nano-)particles with O, N ≫ Mg, Si > S ≫ P, in order of cosmic abundance, or N, P, O, S > Mg, Si, in order of their chemical affinity for (aromatic) ring systems, perhaps provides a promising framework for further exploration. Although these particles would appear to have many advantages as viable DIB candidates, if they are too big, they may actually be too stable to explain the sensitivity of some of the DIBs to UV radiation.

The number of possible structural isomers for a given chemical composition C_*n*_H_*m*_X_*y*_ is enormous; for species with ≳30 heavy atoms, it is in millions. The key (sub-)structures then probably contain only of the order of tens of carbon atoms because any more than this and the number of isomeric variations would be large and lead to millions of DIBs rather than the observed hundreds. Hence, the key (sub-)structures in the species that produce the DIBs must lie in a somewhat restricted set of chemical configurations.

If the DIB carriers were to be formed by a top-down fragmentation process, say by the photolysis or photo-fragmentation of a-C(:H) nanoparticles, then they are most likely to be cyclic species because these are probably the most stable (sub-)structures that would be released from a-C(:H) nanoparticles during UV photolysis. Alternatively or additionally, viable DIB carriers may be found among the more stable photo-dissociation products of these cyclic species (see §6.5). Furthermore, a top-down process would provide an en route triage that weeds out the less stable structures, such as the olefinic/aliphatic links between the aromatic domains [45–47], which will, in any event, be rapidly photo-dissociated in the diffuse ISM (e.g. [44,45,48]) as discussed earlier.

### 6.3 The size and form of the diffuse interstellar band carriers

The infrared emission bands, often attributed to PAH carriers, are almost ubiquitous throughout the ISM but are not observed in HII regions and supernova remnants, where the carriers are expected to be rapidly destroyed [49–52], nor are they seen in dense molecular clouds where they are most likely accreted onto larger grains [53–56]. In the diffuse ISM, aromatic species with ≲30 heavy atoms (C, N, O, …) will probably rapidly be destroyed by UV photons (e.g. [19]). However, when the dehydrogenation and ionization states are taken into account [12], it appears that only aromatic species with ≳50 ‘heavy’ atoms should be resistant to destruction in the regions of the ISM where the IR emission bands are observed [11]. This seems to be coherent with the observed structure in some DIBs being consistent with the rotational contours of rather small (five to seven heavy atom) [32] gas-phase molecules,^6^ which would then be the photo-dissociation products of large species. In supernova-driven shocks and in the hot post-shock or coronal gas, this minimum size is likely to be significantly larger (e.g. as large as particles with hundreds of atoms, [49–51,58,59]) and so DIBs would not be expected in these energetic regions.

Based on experimental results, it would seem that if the DIB carriers are stable species, they must contain ≳40±10 heavy X atoms because lighter species are rapidly destroyed in the ISM (e.g. [19,12]). On the other hand, if they are smaller than this, then the DIB carriers can apparently only be transient species, or must be particularly stable species (see following §6.5), which means that their chemistry and their abundances will be sensitive to the strength and intensity of the local ISRF, particularly to stellar UV photons. Hence, it would seem that we can naturally divide the DIB carriers into two distinct classes:

(i) large, partially dehydrogenated, radical cations resistant to UV photo-processing; C_*n*_H${\;}_{x}^{p+}$ (n≳40, *x*≤(2*n*+1), *p*=0, 1, 2), and
(ii) transient small, highly dehydrogenated, radical cations sensitive to UV radiation or particularly photo-dissociation-resistant species; C_*n*_H${\;}_{x}^{p+}$ (n≲40, *x*≪2*n*, *p*=0, 1, 2).

In the diffuse ISM, it is likely that any pendant methyl, alkyl (C_*n*_H_(2*n*−1)_) or other side groups, such as those seen in the asphaltenes, would be removed through the effects of photo-dissociation/photolysis but that bridging species ought to be more resistant. In the cracked petroleum residues, very large side groups are removed (but nevertheless some remain) and are therefore under-represented in the asphaltene analyses. In the following, we therefore concentrate only on the core aromatic-rich carbon structures and their likely photo-dissociation products.

### 6.4 The likely chemistry of the diffuse interstellar band carrier (sub-)structures

In terms of the possible chemical species that could be the carriers of the DIBs, we would need to explore (experimentally and theoretically) many millions of possible structures (see §2). As an illustration of this, the asphaltene moieties in table 3 show the very rich variety and complexity of aromatic species with only around 20–50 carbon atoms (and no two are the same). However, an unguided search for viable DIB carriers (among the many millions of such large molecules) would be a formidable and inherently intractable task, almost infinitely more difficult than ‘looking for a needle in a haystack’. This is why we have not yet been able to elucidate the fundamental nature of the DIBs carriers, with the exception of C${\;}_{60}^{+}$ and its four associated DIBs [4,5]. The clearly difficult but fruitful experiments that eventually led to the association of C${\;}_{60}^{+}$ with two DIBs were, nevertheless, aided by the fact that fullerenes have been detected in the ISM (e.g. [60]). In this case, the ‘haystack’ to search was known and is rather small. Given that, other than fullerene, no similarly large complex molecule, ion or radical has yet been identified in the ISM, the problem is, in general, that of finding the ‘haystack’ before a search for the ‘needle’ can begin.

Given the above-discussed aromatic-rich domain characteristics gleaned from the analysis of asphaltenes, we can conclude that in the ISM the aromatic-rich domains and sub-domains within a-C(:H) nanoparticles and sub-micrometre-sized grains are most probably:

— relatively compact, with central S_*n*_ cores with *n*=4–10,
— have mixed S_*n*_P_*m*_ structures with one or several peripheral or bridging P rings (*m*≥1),
— include P rings with common N, O, S, Si, P or B hetero-atoms and methylene bridges (–CH_2_–), and
— are inter-connected by alkyl bridges.

These asphaltene-type structures resemble the likely (sub-)constituents of interstellar a-C(:H) (nano-) particles [22,37,44,45,48], which are probably also the type of particles at the heart of fullerene formation in circumstellar regions [61,23].

Based on the structures of the analysed PA and CA asphaltenes (table 3), it is here suggested that an experimental and theoretical search for DIB carrier families could be most usefully directed towards ionized and dehydrogenated 〈H]S_*n*_P^X^S${\;}_{m}^{+}$ species with *n*>1, *m*≥0 and nitrogen-doped hetero-cyclic P rings (i.e. X = N). Here the precursor 〈H] is used to indicate a dehydrogenated state. In the follow-up work, more complex structures, such as 〈H]S_*n*_P^X^P^X^S${\;}_{m}^{+}$ and 〈H]S_*n*_P^X^S_*m*_P^X^S${\;}_{p}^{+}$, where X=N, O, S, Si, P, B, Al, Ge, …might also be worth exploring.

Based on all of the above discussions, other possible DIB-carrying structures could include: cumulene-containing aromatics or cumulene-type rings without or with hetero-atoms (principally nitrogen atoms), the family of couronenes, couronanes and courone-anes (also possibly doped with N).

### 6.5 The stability of the diffuse interstellar band carriers

The inferred DIB carrier size limitation ($N_{\mathrm{heavy}\;\mathrm{atom}}≳40\pm 10$) discussed above is predominantly based on studies of the photo-excitation of PAHs [19] and observations of reflection nebulae [62]. However, there are other photo-stability criteria that can be used to estimate the size and the nature of the DIB carriers and that perhaps place lower limits on the number of heavy atoms per carrier. Independent of size, perhaps the most important criterion is whether, following the stochastic absorption of a UV photon from the interstellar radiation field, a highly excited species can shed the absorbed energy on a time-scale that is shorter than that for photo-thermo dissociation [63], i.e. via C_2_ or some other fragmentation loss processes. Energy loss from an excited state of a DIB carrier may include IR to optical photon emission or reversible structural re-arrangements.

**Figure 9.**
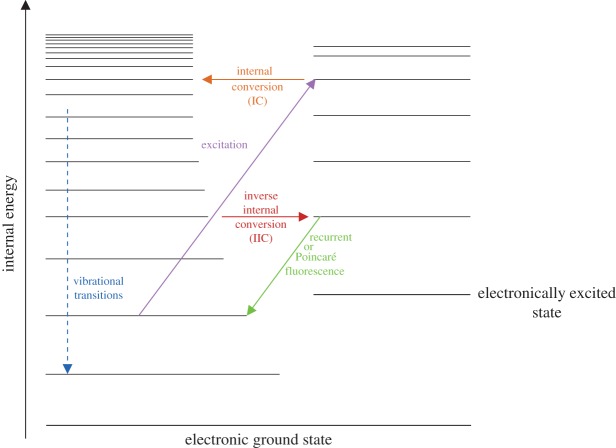
A Jablonski diagram showing the possible transitions for species with low-lying electronically excited states. In general, the electronic ground state is a singlet (spin = 0) and the low-lying electronically excited state a triplet (spin = 1).

Let us first consider energy loss processes via photon emission, which are summarized in figure 9. After excitation by a far-UV photon, the carrier is left in an excited electronic state from which it can shed energy via the relatively slow process of internal conversion (IC) followed by vibrational mid-IR photon emission. In some cases, the carrier can lose the energy by the faster process of inverse internal conversion (IIC) and then the emission of one or more optical photons, involving the process known as inverse electronic relaxation [64], inverse fluorescence [65] or Poincaré fluorescence [66]. For this fluorescent emission to be an efficient cooling mechanism the carrier must have a low-lying electronic excited state, as seen experimentally for C${\;}_{6}^{-}$ [65,67,68] and C_6_H^−^ [68]. Low-lying electronically excited states are also characteristic of defective graphene, C_60_ anions, PAH cations and polyenes, probably making such species resistant to photo-dissociation in the harsh environment of the diffuse ISM. The above indicates that there may indeed be small species, with significantly less than 40 heavy atoms, that are resistant to photo-dissociation as has been inferred for the carriers of the DIB [32,69,70] and the extended red emission (ERE) [71].

**Figure 10.**
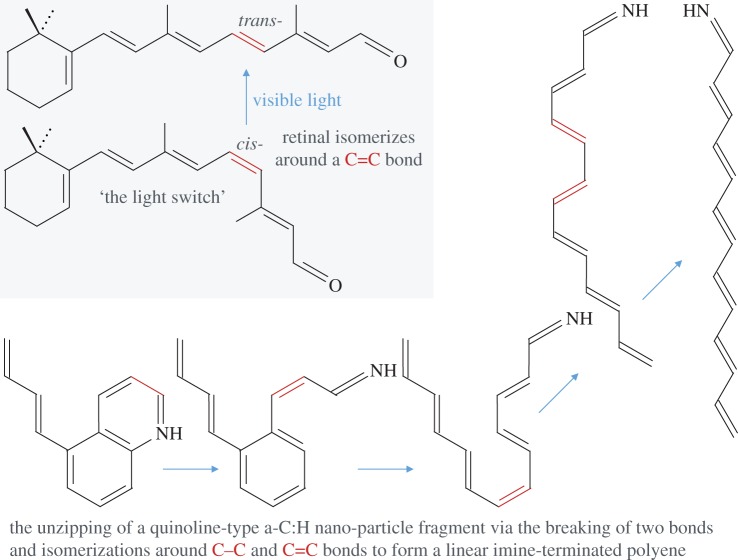
The mechanics of the *cis*- to *trans*-isomerization of the molecular ‘light switch’ retinal (upper left grey box). A schematic view of the possible evolution of a fully conjugated, N-substituted hetero-cyclic (quinoline), a-C:H nanoparticle C_14_H_*m*_ photo-fragment (bottom and right) from an aromatic/olefinic species (left) to an imine-terminated polyene (right).

Now we turn our attention to the less well-known but equally interesting case of molecules that absorb the energy of a UV photon and use this to switch their structure [72]. Among these molecules are linear polyene-based structures, at least four carbon atoms long (i.e. –C=C–C=C–), with methyl side-groups and chain-terminating groups such as =O, cyclohexene and benzene ring derivatives. Polyene structures are typical of well-known molecules, such as the carotenoids *β*-carotene and lycopene, azobenzenes and retinal, the molecule at the heart of vision (see upper left box in figure 10). Upon photon absorption, all of these molecules flip their structure by rotation around a central alkene C=C bond in their conjugated polyene chains, which can be up to approximately 16 C atoms long, i.e. [C=C–]_2*n*_ where *n*=2–8. The structural flip in these species, due to *π* bond breaking and re-formation, is generally reversed by the absorption of lower energy blue photons. Thus, most of these polyene-based species are coloured and may, therefore, be of interest as DIB carriers [73–76]. However, we first need to explore how and why polyenes might form, in particular, in the diffuse ISM.

In the tenuous ISM, UV-EUV photons will progressively photolyse and fragment a-C(:H) nanoparticles into their aromatic and olefinic/aliphatic sub-structures [22,37,44,52]. Some fraction of these fragments could undergo further bond breaking and re-arrangement to form stable polyene-type species, following UV-EUV photon absorption by the fragments.^7^ A schematic view of a nitrogen-substituted olefinic/aromatic a-C(:H) nanoparticle fragment evolving towards a hetero-atomic polyene in shown in figure 10. In this evolution, only two bonds are broken, the first at a polarized carbon–nitrogen bond associated with the more electronegative nitrogen atom and the second at a bridging carbon–carbon bond likely to be a point of weakness in a highly vibronically excited state. Polyene species, with N, O, S, P, … hetero-atoms, will probably exhibit significant permanent dipole moments (μ≳2 D). Thus, polyene species appear to have many interesting DIB-relevant properties and could be particularly viable DIB carrier candidates [73–76] because they

(i) have low-lying electronic excited states, making them stable against photo-dissociation,
(ii) have tuneable properties, e.g. length and double-bond conjugation sequence,
(iii) can incorporate hetero-atoms, e.g. =O in retinal and –N=N– in azobenzene,
(iv) can absorb UV photons and undergo structural switching or flipping,
(v) absorb in the optical and UV and are therefore coloured,
(vi) have a wider isomeric variety than aromatics or polyynes (for a fixed number of C atoms),
(vii) could arise from the photo-processing of aromatic moities,
(viii) will probably dehydrogenate to form (cyano)polyyne species,
(ix) have, to date, received relatively little attention theoretically or experimentally [76].

**Figure 11.**
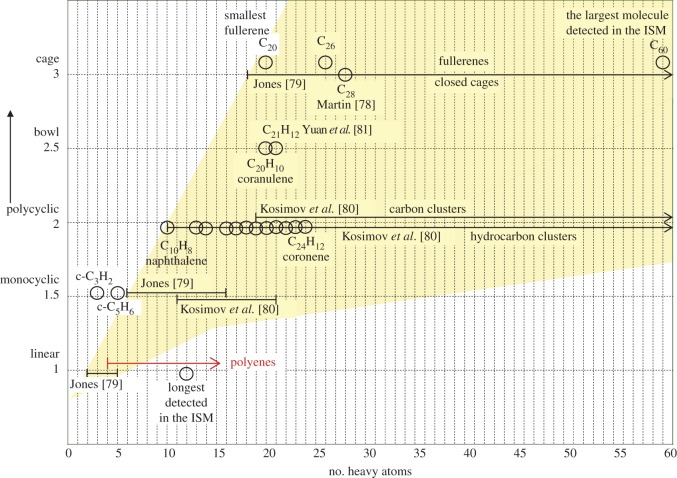
A visual summary of the stability of carbon clusters in terms of their structure and shape taken from the cited references, horizontal black lines, and C_21_H_12_ and C_28_ clusters [78–81]. The horizontal red line shows the range of possible polyene sizes discussed in §6.5.

Thus, it appears that molecular, ionic and/or radical polyene-based structures, which could naturally arise from the degradation of the photo-dissociation products of a-C(:H) nanoparticles, might provide interesting and promising DIB-carrier candidates. Figure 11 compares the stability range for polyenes (red rightward arrow) to those for cyclic molecules, polyynes, PAHs, fullerenes and other carbon clusters as a function of shape [78–81]. This figure shows that these quasi-linear species appear to occupy the same sort of size regime as the linear (cyano-)polyynes. However, as pointed out above, they have a much wider range of substitutional and configurational forms than the polyynes and polycylic aromatics for the same number of carbon atoms. Furthermore, as figure 10 (bottom and right) shows, the polyenes exhibit a wide range of mixed *cis*-/*trans*-isomeric forms, e.g. saw-tooth (*trans*-only C=C), bay (adjacent paired *cis*-forms, C=C–C=C) and mixed saw-tooth/bay structures (mixed *cis*-/*trans*-C=C sites), and each will respond to certain photon energies that can drive particular bond switching transformations (i.e. between *cis* and *trans* conformations) around C=C bonds.

### 6.6 The abundance and lifetime of the diffuse interstellar band carriers

For a molecular/radical/ionic species with a given number of heavy atoms, the abundance of any likely DIB carrier is clearly independent of the number of possible isomers which, as mentioned above, can be more than a thousand (million) for species with 6 (30) heavy atoms. However, the abundance of a viable DIB-carrying isomer will clearly depend upon its stability against photo-thermal dissociation, as discussed above. Other than C${\;}_{60}^{+}$, we currently have no idea as to what are the most likely and most stable species responsible for the DIBs. Thus, it is not yet possible to give a reliable estimate of the likely abundance of the DIB carriers. Nevertheless, if we assume a top-down route to their formation via the fragmentation of nanoparticles, we can provide a ball-park estimate. For instance, the THEMIS model [37,82,83] uses about 100 ppm of carbon in its small a-C(:H) nanoparticle dust component. If we assume that the DIBs were to arise in these grains, this gives an upper limit to the DIB relative abundance of about 10^−6^ with respect to hydrogen, if each nanoparticle contains of the order of 100 carbon atoms. For a lower limit, we can assume that if approximately 1% of the carbon atoms in the nanoparticles (i.e. approx. 1 ppm) were to be liberated into the gas and to form stable DIB carriers, each containing 10 C atoms, this would yield a carrier abundance with respect to hydrogen of 10^−7^. Thus, for a line of sight with a column density *N*_H_∼10^21^ cm^−2^, the DIB carrier column density could, at most, be of the order of *N*_DIB_∼10^14^ to 10^15^ cm^−2^. An estimate of the abundance of the recently identified C${\;}_{60}^{+}$ DIB carrier indicates a column density of ∼2×10^13^ cm^−2^ [5] along the diffuse ISM line of sight towards HD 183143 (*N*_H_∼6×10^21^ cm^−2^), equivalent to a relative abundance of ∼3×10^−9^ or 0.2 ppm of carbon in the form C${\;}_{60}^{+}$, which is significantly lower (by a factor of 30–300) than the above upper limit estimate for the column density of generic DIB carriers. Thus, it seems that the formation of stable DIB carriers by top-down formation from nanoparticles need not be very efficient as long as the carriers are resistant to photo-thermal dissociation in the diffuse ISM. If indeed the C${\;}_{60}^{+}$ carrier of four of the ≳400 DIBs requires 0.2 ppm of carbon, and this is typical of DIBs, then approximately 20 ppm (approx. 6%) of interstellar carbon [84] must be locked up in the carriers, equivalent to approximately 10% of the carbonaceous dust mass [37]. It is likely that the carbon, and the other heavy atoms, tied up in the DIB carriers are not an entirely independent population because the DIBs do, at some fundamental level, correlate with observed dust properties, e.g. extinction and emission, showing weak (anti-)correlations with *E*(*B*−*V*), the UV bump and FUV extinction. Thus, the required carbon, and other heavy atoms, in the DIB carriers is most probably not an additional abundance requirement but must, at least in part, be included within the required dust elemental depletion budget, i.e. within some dust component whose composition, structure and properties we do not yet fully understand.

Recent work [52] suggests that the lifetime of a-C(:H) nanoparticles against photo-thermal dissociation and di-cationic Coulomb fragmentation appears to be $≳10^{6}-10^{8}$ yr in the diffuse ISM. Thus, if the DIB carriers are the products of the top-down fragmentation of nanoparticles, then this is also their replenishment time-scale. In the diffuse ISM, the DIB carriers must then have lifetimes ≥10^6^−10^8^ yr, which appears to be consistent with that expected for large molecules under the PAH hypothesis. In contrast, under the harsh radiation field conditions within HII region cavities, the nanoparticle di-cationic Coulomb fragmentation time-scale is only a year or so. Hence, the DIB carriers are not be expected to survive inside HII region bubbles and therefore by inference within hot supernova bubbles and supernova shock waves, as per PAHs [49,50] and small a-C(:H) grains [51,58,59].

### 6.7 Diffuse interstellar bands and chemical associations

An association between certain DIBs and small radical species, e.g. C_2_, C_3_, CH, CN, OH, CH^+^ and OH^+^, is supported by the observational evidence, but an interpretation of the correlations, anti-correlations or non-correlations is far from straightforward. Nevertheless, strong DIBs do seem to trace the diffuse atomic gas (e.g. [85]) but only the 619.60 and 661.36 nm DIBs appear to show a nearly perfect correlation across numerous lines of sight, covering a wide range of environments [28]. Among the di-atomic radicals, there does appear to be a general correlation between C_2_, CN, CH and OH, but these species do not seem to correlate with CH^+^ and OH^+^ (e.g. [86]). The so-called C_2_ DIBs, which are relatively weak and seemingly associated with denser gas, also appear to correlate with C_2_, CN and CH [87]. Furthermore, a number of studies seem to show that some DIBs correlate better with CH than with CN, with the C_2_ and C_3_ rotational temperature or with the C_2_ column density, while others anti-correlate with a high CN rotational temperature [24,25,30,86]. There is also evidence that some DIBs appear to be weaker towards regions where strong CN absorption is observed, e.g. towards the supernova SN2014J in M82 [33]. However, in all of these works, only a relatively limited number of DIBs were studied and so it is hard to generalize, particularly so because the correlations are made using a mix of observed equivalent widths and column densities. The correlation issue is perhaps somewhat clouded by the fact that both gas and dust are traced by the total hydrogen column density and so will always correlate to some degree or another. Thus, the question of DIB families is still an open one, but the idea of two broad classes does appear tenable, i.e. (i) broad and strong DIBs associated with weak molecular features and (ii) weak and broad DIBs associated with the strong lines of simple radicals [88]. It is, therefore, difficult to make any firm inferences about the relationship between the DIBs, small radicals and the extinction due to interstellar nanoparticles. However, and within the framework of a top-down mechanism for the formation of the DIB carriers via nanoparticle fragmentation, it is perhaps possible to generally conclude that

(i) the carriers of the ‘C_2_’ DIBs are seemingly associated with or formed in denser gas,
(ii) the anti-correlation of some DIBs with CN could be taken to imply that nitrogen-containing hydrocarbons play a role in the DIB carrier chemistry, i.e. when DIB carriers are destroyed, nitrogen appears in the gas as CN and the DIBs are weak or absent,
(iii) a weak UV bump and steep UV extinction can result from the accretion of aliphatic-rich, a-C:H mantles on grains in denser regions [45,37,56,89], but weak DIBs are not necessarily a consequence, unless the DIBs only result from aromatic-rich a-C materials,
(iv) a weak UV bump and steep UV extinction is also possible in energetic regions, as a result of nanoparticle destruction (e.g. [49,51]), leading to weak or absent DIBs, and
(v) the DIBs seemingly do not correlate with small cation radicals (CH^+^ and OH^+^) and therefore the carriers are likely to be disfavoured in highly excited regions.

An observable link between nitrogen and the DIBs will probably be rather difficult to elucidate because its depletion is very low, rather invariable and it does not seem to follow that of the other elements in the transition to denser regions [90]. In the diffuse ISM, the dust does not appear to be very rich in nitrogen-bearing species; for instance, observations indicate that there is ⩽0.3% of the available nitrogen in –C≡N bearing organics [91] and so nitrogen can, therefore, only be a trace dust species, albeit a potentially very important trace element. Nevertheless, nitrogen does appear to be an important dopant in organic nano-globules [17,92–95].

### 6.8 The diffuse interstellar bands and the extended red emission

It is likely that the DIB carriers are related to the ERE carriers and that both arise in species that are marginally stable in a wide variety of environments, particularly in the diffuse ISM [96]. The DIB and ERE originators must both involve carriers with electronic transitions 1.4–2.2 eV above a ground state [96]. Interestingly, electronic transitions in the range 1.4–2.2 eV above a ground state look remarkably similar to the 1.0–2.6 eV optical gaps typical of hydrogenated amorphous carbon materials, a-C:H (e.g. 1.0–2.7 eV [97]). As has also been pointed out, the DIB and ERE carriers show non-unique global spectra, perhaps indicative of broad families of particles that are sensitive to the local environment, especially the UV radiation field [96].

A particularly interesting region for ERE observations is the Red Rectangle region, which shows an unusual geometry with both internal and external (interstellar) sources of UV photons [98]. Here, the ERE occurs only along the walls of an outflow cavity where there is an unobscured view of the FUV photons from the central stars [99], but a blue luminescence (BL) is seen only from those regions that are shielded from the interior FUV radiation [100,101]. It appears that this geometry is rather unique and allows for a study of the UV photon-driven evolution of a-C:H grains, from wide to narrow band gap materials, and could lead to the formation of viable (precursor) DIB carrier candidates [44,102]. Nevertheless, only two weak DIBs (578.0 and 661.3 nm) have been observed in absorption in the Red Rectangle region itself or in an intervening diffuse interstellar cloud [103]. It, therefore, seems likely that this region probably forms the DIB precursors, but that the actual DIB carriers are produced only by further UV photo-processing in the low-density diffuse ISM. As was pointed out, in relation to the Red Rectangle region, the hetero-atom doping of a-C:H materials may provide a rather interesting link between the DIBs, ERE and BL [48,44]. Particularly interesting is the connection between the BL, nitrogen-doped a-C:H materials and a 442.8 nm band at the wavelength of the most prominent DIB.

Another unusual and interesting object of key relevance to our understanding of the DIBs is Hershel 36, which is associated with a triple O star system in the M8 HII region with an elevated visible/UV stellar radiation field and a flat UV extinction (*R*_*V*_∼6). This object exhibits relatively weak DIBs, sits behind a dark lane with *A*_*V*_∼4 mag. and *N*(*HI*)=8.9×10^21^, shows (non-thermal) rotationally excited CH and CH^+^ and vibrationally excited H_2_, due to strong IR radiation from an adjacent source but no CN and C_2_ [32,69,85,104]. Along this line of sight, the DIBs are broad and many (approx. 25%) show extended red wings [32,104]. This is probably another region where, as per the Red Rectangle, dense cloud carbonaceous dust is being actively UV-processed to form DIB carriers. However, unlike the Red Rectangle, Hershel 36 clearly shows DIBs, but DIBs that are somewhat different from those observed elsewhere.

In the following section, we explore a top-down fragmentation scenario for the formation of the DIB carriers.

## 7. Top-down branching and diffuse interstellar band carrier formation

While no two individual interstellar nanoparticles are likely to be identical, the population as a whole will probably incorporate the sub-structures that actually give rise to the electronic transitions that are responsible for the DIBs. These sub-structures within nanoparticles will be protected by the surrounding material and the associated transitions would likely give rise to broader DIBs that seem to be more widely distributed spatially and that are relatively stable against UV (e.g. [85]). However, in a low density ISM with a hard UV radiation field, the nanoparticles will be partially dehydrogenated (e.g. [22]), ionized and thus rendered more susceptible to dissociative fragmentation processes (e.g. [12]). Their daughter fragmentation products are likely to be aromatic-rich moieties, and the DIBs that these sub-nm fragments may carry would be UV-sensitive and formed in the ISM by the top-down fragmentation of hetero-atom doped a-C(:H) nanoparticles. These fragments would then themselves be fragmented into non-DIB-carrying small polyatomic radical species C_2_, C_3_, CN, CH, CH^+^, OH, …) and eventually their constituent atoms/ions (C, C^+^, N, N^+^, O, S, Si, P, …). The rate of this top-down fragmentation process will be determined by both the intensity and hardness of the ambient UV radiation field and will lead to a steep sub-nanoparticle fragment size distribution with a steepness that depends upon the photo-dissociation rate of all sub-nm species. This may explain why the same DIBs are sometimes seen with and without the same molecular radical species, with a much harder and/or more intense UV radiation field being present in the without case.

**Figure 12.**
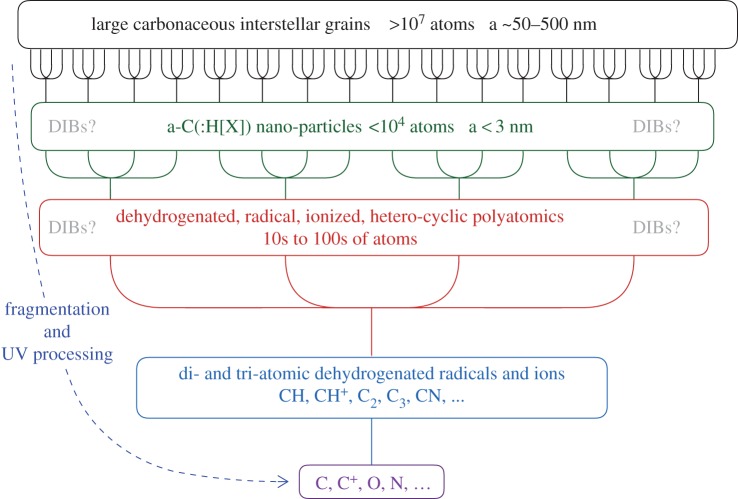
A schematic view of the hierarchal top-down route to the formation of the DIBs carriers by fragmentation and their formation, evolution and destruction by UV photo-processing.

Here a top-down branching scenario (figure 12) is proposed for the formation of the DIB carriers. Top-down branching is like tree branching, but the sequential evolution is from top to bottom with chemical variety decreasing from top to bottom in line with a decreasing chemical complexity and decreasing number of constituent heavy atoms. At the top of the tree are the large grains, in the middle branches the nanoparticles, in the lowest branches and upper trunk can be found the molecules and at the very base of the trunk the atoms and atomic ions.

In this scheme, the number of configurations is reduced to well below that of all possible configurations, by processes that leave only the most stable fragments. Hence, descending in size through the effects of fragmentation leads to a more limited number of smaller fragments with a more restricted conformational range.

### 7.1 Tree tops: big grains

It has been suggested that in the ISM the big carbonaceous grains, composed principally of a-C(:H), are probably the source of all of the smaller carbon-rich grains [45,37]. However, it is difficult to imagine that these grains can consist of only carbon and hydrogen atoms as they must, at their sites of formation (around evolved stars and via accretion in molecular clouds) and during their sojourn in the ISM, become polluted with hetero-atoms, i.e. they are actually a-C:H:X grains doped with X hetero-atoms [48,44] and/or carbonyl-rich functional groups perhaps not unlike the meteoritic and cometary organic nano-globules [17]. This pollution must be carried down into the smaller grains in the diffuse ISM through the effects of fragmentation (e.g. [37,45,105]). These polluting hetero-atoms and groups will be incorporated into the grain chemical structure resulting in interesting functional groups and structures, many of which are likely to have electronic transitions lying within the visible wavelength range. This scenario, developed in the following section within the framework of nanoparticles, would quite naturally explain why the DIBs generally correlate with the interstellar extinction, as measured by *E*(*B*−*V*), and the continuum thermal emission from dust.

It is now clear that the dust in the ISM does vary (in structure and composition) from one region to another (e.g. [37,83,106]) and so a region-to-region environmental dependance of the DIBs is naturally to be expected. For instance, and if the DIBs are, as we propose below, due to nano- particles and smaller, then there is a clear environmental explanation as to why they do not associate with the molecular hydrogen in the denser interstellar gas. This is due to the evolution of the dust properties in these regions where the dust has accreted mantles of a-C:H and has been coagulated into aggregates. Here all the small DIB-carrying nanoparticles have been swept up into larger structures in which their electronic transitions are submerged by the global, aggregate dust optical properties.

### 7.2 Middle branches: nanoparticles

**Figure 13.**
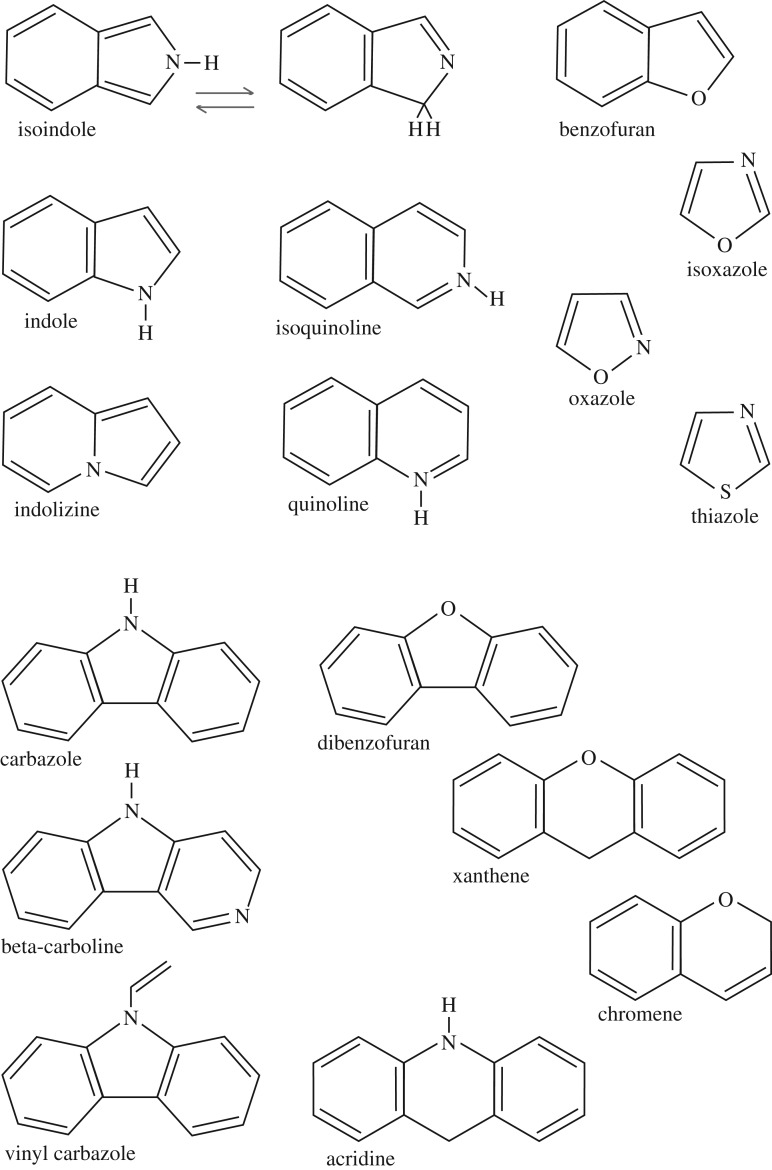
Hetero-cyclic species (P^XX^, SP^X^, S${\;}_{2}^{X}$, SP^X^S and S${\;}_{3}^{X}$, with *X*=*N*, O or S) with 5–15 heavy atoms and interesting DIB colour-related properties, including that: many of them are coloured, they and their derivatives are used in dye-making and/or photo-receptors, some of them fluoresce when exposed to UV and all are stable species widespread in nature.

Hetero-cyclic sub-structures are widespread in nature in aromatic moieties and their existence in interstellar carbonaceous grains is, therefore, almost a certainty [48,44]. Their presence in interstellar nanoparticles is, therefore, practically unavoidable and they are likely to be at the heart of the more stable and UV-resistant DIBs observed in the ISM. Figure 13 shows some of the many possible hetero-cyclic molecular structures, including only N, O and S hetero-atoms, which are of interest to the DIB problem because many of them are coloured, used in dye-making/photo-receptors and some of them fluoresce when exposed to UV. However, hetero-cyclic structures with phosphorus, boron and silicon hetero-atoms are also well known and therefore equally likely to exist within the interstellar dust population. The structures shown in figure 13 are all well-known and distinct molecules; however, in the diffuse ISM, they can probably only exist as sub-structures within larger (nano-)particles where they would be protected from the dissociating effects of the interstellar UV radiation field, i.e. they would probably be less dehydrogenated and more neutral than ionized. These structures could perhaps therefore explain the broader DIBs that are more resistant to UV radiation DIBs.

If interstellar nanoparticles are indeed responsible for some of the DIBs, then their intrinsic electronic transitions would naturally be broadened due to the perturbations introduced by their immediate environment within the larger grain structure. Thus, the responsible electronic transitions are intrinsically broader than any atomic or molecular lines.

Given that the hetero-cyclic structures likely to be at the heart of the DIBs will undergo a top-down processing, structurally related DIBs will form one from another. For example, in figure 13, it can be seen that adding or removing rings from some of the structures leads to their inter-conversion, e.g. the molecules *β*-carboline or carbazole are so related to the smaller isoindole and pyrrole molecules (the latter a fivefold ring with one nitrogen hetero-atom is not shown) and similarly dibenzofuran and benzofuran, and also xanthene and chromene. Hence, it is not unexpected that inter-DIB correlations do not always go through zero because the formation of some DIB structures will probably depend upon the destruction or transformation of others. Furthermore, that the DIBs generally do not correlate well with each other is probably due to the wide variety of possible structures that can give rise to electronic transitions at the wavelengths of interest for the DIBs.

No significant correlation between the UV bump, the far-UV extinction rise and the DIB strengths has been found (e.g. [107]) However, in the Small Magellanic Cloud, it appears that when the UV bump is weak or absent, so are the DIBs [108]. Thus, there is some evidence that when the interstellar nanoparticles responsible for the UV bump are destroyed, so are the DIB carriers and, as noted above (§6.7), the anti-correlation of some DIBs with CN seems to indicate that nitrogen may play a role in the DIB carrier chemistry. Hence, by inference, it would appear that nitrogen could be an important hetero-atom dopant (at the % level) in interstellar hydrocarbon nanoparticles.

### 7.3 Low branches: radical and molecular ions

The disruption of interstellar nanoparticles, most probably driven by UV photon processing (through photo-dissociation and charge effects), will yield smaller fragments that are then even less stable in the hard UV radiation field than are their parent grains. In this case, the liberated species will now look more like molecules than nanoparticles but must obviously suffer the consequences and bear the marks of dehydrogenation and exist as cations in these harsh environments (e.g. [107]). Nevertheless, the observed structure in DIBs that appear to be somewhat UV-resistant does seem to be consistent with the rotational contours of rather 5−40 atom gas-phase molecules [57,32].

**Figure 14.**
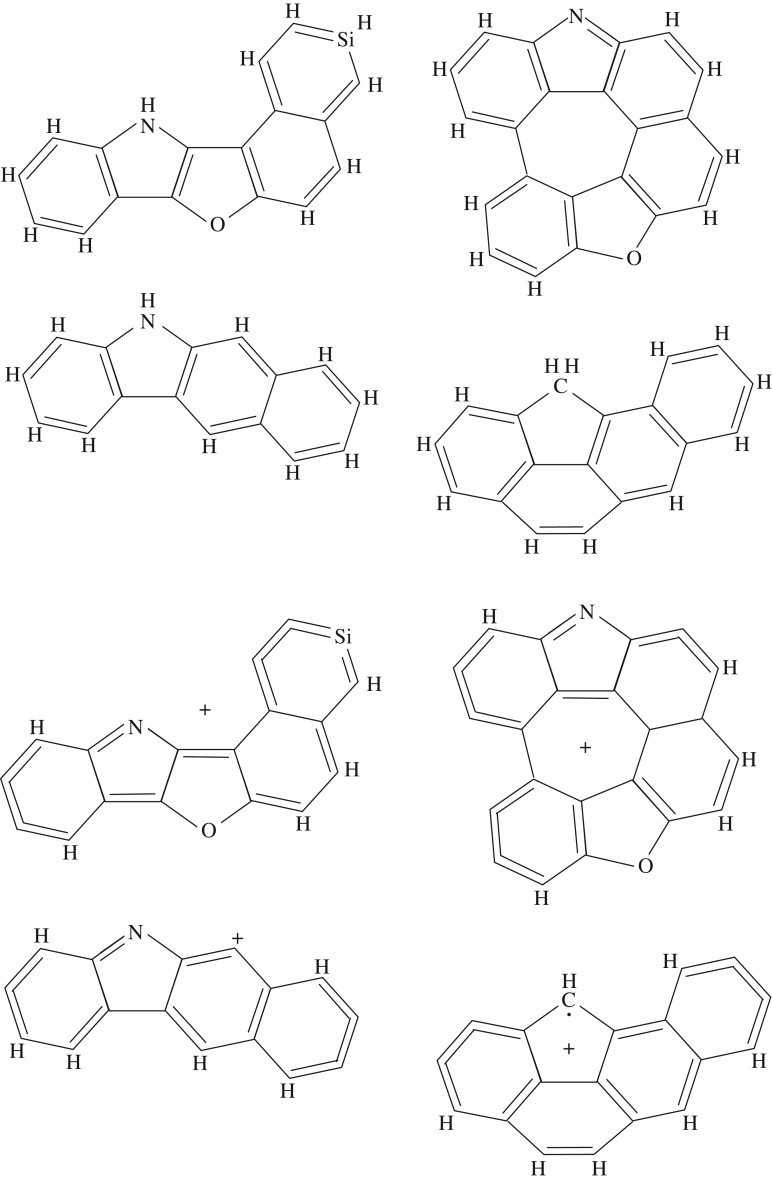
Four likely DIB precursor molecules, with 17–24 heavy atoms and the following structures S_2_P^O^P^N^S, S_2_P^O^SGSP^N^, S_4_P′ and S_2_P^N^S. The lower part of the figure shows their ionized and ≈50% dehydrogenated forms, 〈H]S_2_P^O^P^N^S^+^, 〈H]S_2_P^O^SGSP^N^^+^, 〈H]S_4_P′^+^ and 〈H]S_2_P^N^S^+^: such polycyclic, hetero-cyclic, radical cations could be candidate DIB carriers.

**Figure 15.**
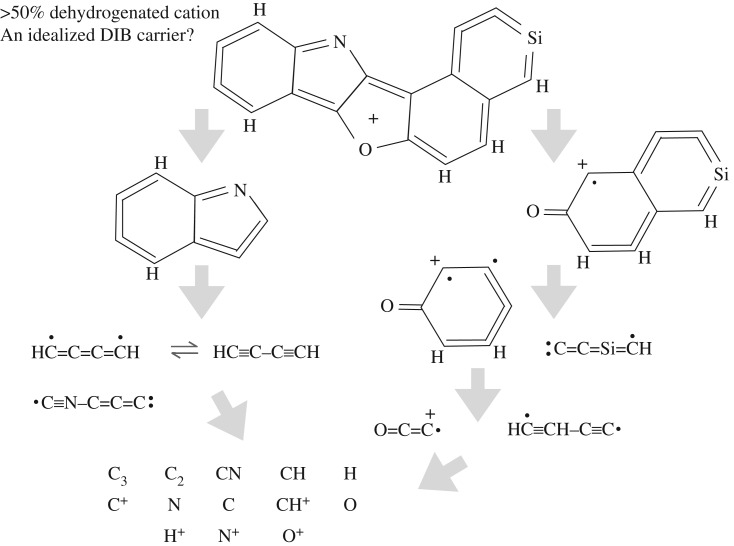
A schematic scenario for a top-down evolution of the DIB carriers. Note that the proposed DIB carrier, 〈H]S${\;}_{2}^{\mathrm{Si}}$P^O^P^N^S^+^, is shown with unrealistically abundant hetero-atoms (N, O and Si) in order to illustrate the possible evolutionary pathways that will need to be explored in more detail.

The upper four hetero-atomic, polycyclic species in figure 14 (C_17_H_11_NOSi, C_22_H_10_NO, C_19_H_12_ and C_16_H_11_N, clockwise from top left) with S_2_P^O^P^N^S, S_2_P^O^SGP^N^S, S_4_P′ and S_2_P^O^P^N^S structures are some possible DIB precursor species, which are shown as rather hetero-atom rich (e.g. N, O and Si containing) for illustrative purposes only. These structures are all viable, hetero-cyclic aromatic moieties, having less than 30 heavy atoms, that would not be stable against UV photo-dissociation in the diffuse ISM. The likely DIB-carrying forms of these species, their ≈50% dehydrogenated cations, are shown in the lower part of the figure. These types of aromatic, radical, cation, moieties will also be dissociated by UV photons in the diffuse ISM but could represent the sort of top-down transient fragments that therefore carry the UV-sensitive DIBs. A schematic scenario for the likely top-down evolution of the DIB carriers is shown in figure 15, where the proposed DIB carrier is illustratively shown with N, O and Si hetero-atoms, corresponding to about a 14% (3/21) heavy atom doping level.

Given the discussion in the previous sub-section, it is most probable that nitrogen with its intrinsic propensity to incorporate into aromatic rings, and especially fivefold rings, will be the most important aromatic cycle hetero-atom. Nitrogen readily inserts into these structures and is known to form a myriad of interesting organic molecules, such as pyrroles, pyridines, pyrimidines, azoles, indoles and quinolines. However, dihetero-atomic cycles could perhaps play a more important role because it appears that many of them exhibit many colourful properties, especially species with two nitrogen atoms (e.g. imidazole and pyrimidine) or a nitrogen and an oxygen atom (e.g. oxazoles).

### 7.4 The trunk: di- and tri-atomic radical ions

The photo-dissociation of the radical and molecular ions discussed in the above sub-section would quite naturally lead to the formation of di- and tri-atomic radical ions, such as CH, CH^+^, CN, C_2_ and C_3_, which appear to bear some relation with the observed DIBs. For instance, the observation of the same DIBs with or without these same radicals in different regions of the ISM could be explained by variations in the local UV radiation field, which would determine the rate of photo-dissociation and therefore how quickly the nanoparticle fragments are photo-dissociated into smaller radical species, e.g. C_2_, C_3_ and CN, which are then themselves photo-dissociated into their constituent atoms and ions C, C^+^, N, etc.

In particular, weaker DIBs appear to be associated with strong CN absorption (e.g. [33]), which would be natural if some of the DIBs are due to the type of nitrogen-containing moieties proposed above and whose end-of-the-line photo-dissociation/fragmentation products would then naturally include CN. Also, the observation of CN with a high rotational temperature in an interstellar cloud that appears to exhibit no DIBs [30] could be explained by the very rapid destruction of the DIB carriers to their excited di-atomic and atomic/ionized constituents. Once again supporting the view that nitrogen is an important (hetero-atomic) element for the DIBs.

### 7.5 The base: the atomic/ionized interstellar medium

At the base of the tree, and in the most diffuse ISM where molecular radicals are rapidly UV photo-dissociated, we find the isolated atoms and ions in the gas, principally, H, C, C^+^, O, N, Si^+^ and S^+^, some of which were constituents within the dust there. The ultimate product of UV photo-processing is the small polyatomic radicals and ions, such as CH, CH^+^, CN, C_2_ and C_3_. Furthermore, in regions of the ISM where there has been heavy UV processing and where significant dust destruction, especially of carbon-rich nanoparticles, has occurred, then it appears that there are no DIBs, i.e. there can be no DIBs without nanoparticles, but there probably can be nanoparticles without DIBs.

## 8. Experimental and observational considerations

Given that an experimental exploration of the many millions of possible DIB carriers is an intractable problem, it would be best to take a statistical approach and therefore explore numerous possibilities at the same time. Such an approach will allow a considerable narrowing of the search once DIB(-like) transitions have been found in a viable material. With this kind of approach in mind, the following suggestions may prove useful in determining a long-term experimental programme with the aim of finding viable classes of materials that could be, or hopefully are, the DIB carriers, i.e. with the aim of finding the haystacks within which to search for the needles.

It is here suggested that a fruitful experimental search should therefore be directed towards exploring the

(i) Low-temperature NIR-UV spectroscopy of asphaltenes both with and without sample irradiation by FUV photons, which would drive both critical sub-structure dehydrogenation and cation formation.
(ii) Low-temperature NIR-UV spectroscopy of a series of single hetero-atom (X=N, O, S, Si, P, …) doped a-C(:H):X materials both with and without FUV irradiation (as per (i)). Initially, at least, the aromatic-rich and aliphatic-rich near-end members of the a-a-C(:H):X material family should be explored, with a later exploration of the middle ground materials.
(iii) Follow-up spectroscopy under identical conditions (as per (i) and (ii) above) but for mixed hetero-atom (N+O, N+S, N+Si, N+P, etc.) doped a-C(:H):X:Y and eventually triply doped (N+O+S, N+O+Si, N+O+P, …) a-C(:H):X:Y:Z materials.

Once DIB-carrying solid phases have been determined, the relevant carriers should then be subjected to closer scrutiny to locate the sub-structures responsible for the DIBs. Furthermore, this approach could then be combined with the low-temperature visible/near-IR spectroscopy of colour-carrying aromatic moieties, such as those shown in figure 13, both with and without FUV-irradiation, in order to explore the neutral and dehydrogenated/ionized forms.

It also appears that there might be observational avenues that could yet yield something interesting. For example, the relationship of the DIBs with line-of-sight depletions, particularly with that of nitrogen, does not appear to have been extensively studied and this could perhaps be worth a deeper exploration.

## 9. Conclusion

It seems that taking a global view of interstellar dust and gas evolution, within the framework of a top-down fragmentation model, may provide a promising new way to view the nature of the DIBs. The diffuse ISM is indeed a harsh environment and small species, with less than many tens of heavy atoms, do not do well there. Thus, the UV processing of interstellar matter at nm and sub-nm size scales would appear to link together and lie at the heart of many of the dust and gas variations that are observed in space.

The origin of the DIBs almost certainly lies in the hetero-cyclic five- and sixfold ring aromatic moieties that form an integral part of the contiguous structure of interstellar nm and sub-nm sized particles, i.e. they are most likely specific, multi-ring (aromatic), sub-structures within nanoparticles. In particular, the aromatic sub-structures within the nanoparticles will be somewhat protected from the effects of interstellar UV radiation and could explain the more UV-resistant DIB population. However, once these moieties are released from nanoparticles as ‘free-flying’ species, they will be significantly dehydrogenated and will also be ionized to their cationic forms. Such species in the gas phase will be significantly more susceptible to UV photolysis than their neutral and fully hydrogenated counterparts once liberated from their parent nanoparticles. They could thus explain the UV-sensitive DIB population. Thus, it would appear that some significant fraction of the DIB carriers could be ‘unstable’, transient species that are rather sensitive to the local physical conditions.

Significantly dehydrogenated and ionized aromatic moieties are much less stable than their parent hydrogenated neutrals in the diffuse ISM and will not necessarily re-form the original aromatic (PAH) structure upon re-hydrogenation by atomic H from the gas phase. They may rather fragment to form smaller ring systems and/or linear chains. Thus, for many highly dehydrogenated aromatic structures (〈H]PAHs) in the ISM, there may be no way back home to their parental structures upon reaction with gas-phase atoms and ions.

The ideas presented here do not necessarily simplify the experimental or observational search for the exact form of the DIB carriers, for if they were rather simple we would have long ago found the solution to this long-standing astro–chemical–physical problem.

The author hopes that the ideas presented here might help to focus laboratory and theoretical studies along a track that may, on the way to the desired destination, uncover some interesting science and perhaps even elucidate the generic nature of the undoubtedly complex species that are the carriers of the DIBs.

## Footnotes

1 The ideas presented here and in the accompanying papers [16,17] arise from a re-consideration of the nature of interstellar dust within the framework of *The Heterogeneous dust Evolution Model for Interstellar Solids* (THEMIS) [18]. A brief introductory description to THEMIS is given in Paper I [16].

2 Fluorene, C_13_H_10_, phenanthrene, C_14_H_12_, pyrene, C_16_H_10_, perylene, C_20_H_12_, benz[a]anthracene, C_18_H_12_, chrysene, C_18_H_12_, triphenylene, C_18_H_12_, and coronene, C_24_H_12_.

3 A perhaps somewhat analogous structure in a-C(:H) nanoparticles could be the sites for the pairwise elimination of adjacent hydrogen atoms from the aliphatic/olefinic bridges linking the intrinsic aromatic domains, as recently suggested as a viable route to H_2_ formation in moderately excited PDRs [22].

4 Indeed, such a top-down process has often been suggested as a viable route for interstellar nanoparticle, molecule, radical and DIB carrier formation [22,32,36–39].

5 D. E. Welty 2015, private communication.

6 It was previously reported for the same set of DIBs that the carriers must be 20–40 heavy atom gas-phase molecules [57].

7 This in much the same way that some fraction of dehydrogenated and vibrationally excited a-C(:H) nanoparticles in planetary nebula dust shells could form stable fullerene structures [61,23]. Some evidence to support aromatic ring-opening perhaps comes from a study of the decomposition of a triply charged biphenyl cations in which one of the rings opens up to form a C_4_ polyene chain [77].

## Data accessibility

Any data associated with this work are available in the cited bibliography.
